# Do generic utility measures capture what is important to the quality of life of people with multiple sclerosis?

**DOI:** 10.1186/1477-7525-11-71

**Published:** 2013-04-25

**Authors:** Ayse Kuspinar, Nancy E Mayo

**Affiliations:** 1 School of Physical and Occupational Therapy, Faculty of Medicine, McGill University, 3654 Promenade Sir-William-Osler, Montreal, QC, 3G 1Y5, Canada; 2 Division of Clinical Epidemiology, Royal Victoria Hospital, Montreal, QC, Canada

**Keywords:** Multiple sclerosis, Quality of life, Health-related quality of life, Measurement, Utilities

## Abstract

**Purpose:**

The three most widely used utility measures are the Health Utilities Index Mark 2 and 3 (HUI2 and HUI3), the EuroQol-5D (EQ-5D) and the Short-Form-6D (SF-6D). In line with guidelines for economic evaluation from agencies such as the National Institute for Health and Clinical Excellence (NICE) and the Canadian Agency for Drugs and Technologies in Health (CADTH), these measures are currently being used to evaluate the cost-effectiveness of different interventions in MS. However, the challenge of using such measures in people with a specific health condition, such as MS, is that they may not capture all of the domains that are impacted upon by the condition. If important domains are missing from the generic measures, the value derived will be higher than the real impact creating invalid comparisons across interventions and populations. Therefore, the objective of this study is to estimate the extent to which generic utility measures capture important domains that are affected by MS.

**Methods:**

The available study population consisted of men and women who had been registered after 1994 in three participating MS clinics in Greater Montreal, Quebec, Canada. Subjects were first interviewed on an individualized measure of quality of life (QOL) called the Patient Generated Index (PGI). The domains identified with the PGI were then classified and grouped together using the World Health Organization’s International Classification of Functioning, Disability and Health (ICF), and mapped onto the HUI2, HUI3, EQ-5D and SF-6D.

**Results:**

A total of 185 persons with MS were interviewed on the PGI. The sample was relatively young (mean age 43) and predominantly female. Both men and women had mild disability with a median Expanded Disability Status Scale (EDSS) score of 2. The top 10 domains that patients identified to be the most affected by their MS were, work (62%), fatigue (48%), sports (39%), social life (28%), relationships (23%), walking/mobility (22%), cognition (21%), balance (14%), housework (12%) and mood (11%). The SF-6D included the most number of domains (6 domains) important to people with MS, followed by the EQ-5D (4 domains) and the HUI2 (4 domains) and then the HUI3 (3 domains). The mean and standard deviation (SD) for the PGI, EQ-5D and the SF-6D were 0.50 (SD 0.25), 0.69 (0.18) and 0.69 (0.13), respectively. The magnitude of difference between the PGI and the generic utility measures was large and statistically significant.

**Conclusion:**

Although the generic utility measures included certain items that were important to people with MS, there were several that were missing. An important consequence of this mismatch was that values of QOL derived from the PGI were importantly and significantly lower than those estimated using any of the generic utility measures. This could have a substantial impact in evaluating the effect of interventions for people with MS.

## Introduction

Multiple sclerosis (MS) is a chronic disease resulting from inflammation and demyelination in the central nervous system (CNS) [1] that is associated with a variety of symptoms, such as fatigue, impaired mobility and cognitive decline [2]. Several new therapies, behavioural [3-9], medical [10-14], and surgical [15-19], have been developed in the field of MS. As there are both benefits and harms from interventions, the importance of considering the patient’s perspective in the evaluation of these new therapies is increasingly being emphasized. Patient-reported outcomes are used to evaluate the patient’s perspective on the impact of the disease and its treatment on symptoms, function, and other aspects of quality of life (QOL). QOL is defined as an “*individuals’ perception of their position in life in the context of the culture in which they live and in relation to their goals, expectations, standards and concerns *[20].” QOL is a global construct that includes domains other than health such as job satisfaction, quality of housing, and the neighborhood in which one lives [21]. Health-related quality of life (HRQL), on the other hand, is a construct that is narrower and focuses on domains within the purview of the health care system, such as normal ranges for physiological variables, physical, mental and social well-being [22,23]. Health status, a term often confused with HRQL, is a description and/or measurement of the health of an individual or population at a particular point in time against identifiable standards [24].

While there are a common set of domains that are relevant across a wide variety of health conditions, including none, these domains may be affected differentially because of the positive and negative effects of interventions. For example, a treatment may have a positive effect on one domain (e.g. mental health) but a negative one on another (e.g. physical health) and this would be condition and intervention specific.

The most widely used methodology to create an index that weighs gains in one domain against losses in another is based on utility theory. Utility measures (or preference-based measures) provide a single value for the construct (health status, HRQL, or QOL) ranging from 0 (for death or worst possible health state) to 1 (for perfect health or best possible health state) [25-29]. This value is used to calculate what is termed a “Quality-Adjusted Life Year” (QALY) which captures the effect of an intervention on quantity of life (mortality) and “quality of life” (which is conceptualized as morbidity) [30-33]. The “Q” in QALY is a misnomer given it measures only the health aspects of QOL, the other aspects, which have been elegantly identified by Flanagan, are physical and material well-being, relations with other people, social community and civic activities, personal development and fulfillment, and recreation [34].

The three most widely used utility measures, namely the Health Utilities Index Mark 2 and 3 (HUI2 and HUI3), the EuroQol-5D (EQ-5D) and the Short-Form-6D (SF-6D), label the constructs underlying these measures as health status and/or HRQL [35-39]. None list QOL as the construct being measured. Yet, for economic evaluation, the QALY is the parameter calculated and compared with cost.

In line with guidelines for economic evaluation from agencies such as the National Institute for Health and Clinical Excellence (NICE) and the Canadian Agency for Drugs and Technologies in Health (CADTH), these measures are currently being used to evaluate the cost-effectiveness of different interventions in MS. However, the challenge of using such measures in people with a specific health condition, such as MS, is that they may not capture all of the domains that are impacted upon by the health condition. If important domains are missing from the generic measures, the value derived will be higher than the real impact creating invalid comparisons across interventions and populations.

Personalized measures have been proposed as a method for identifying those aspects of a health condition that impact on QOL. While they may differ from person to person and across health conditions, the value derived from them represents QOL. The most commonly used individualized measures of QOL are the Patient Generated Index (PGI) and the Schedule for the Evaluation of Individual Quality of Life-Direct Weighting (SEIQOL-DW). Both measures capture the individual’s perspective on QOL, by permitting him/her to nominate the areas of life that are most important and assign a weight to each domain. Personalized measures of QOL have been used in several clinical trials to evaluate the effectiveness of different interventions on overall QOL [40-44]. Furthermore, these measures have shown to be particularly useful in clinical settings by improving patient-physician communication and by helping prioritize treatment options [45-47].

The global aim of the study is to contribute evidence for the content validity of generic utility measures with respect to capturing the relevant domains for people with MS. The specific objective was to estimate the extent to which generic utility measures capture important domains that are affected by MS.

## Methods

### Subjects

The data for this study comes from a study of the life-impact of people diagnosed with MS during the era of magnetic resonance imaging (MRI) and disease modifying therapies (the New MS) [48]. The available study population consisted of both men and women who had been registered after 1994 at the three participating MS clinics in Greater Montreal, Quebec, Canada. The study was approved by all regional ethics committees. Inclusion criteria for the study were diagnosis of MS or Clinically Isolated Syndrome (CIS) after 1994. From a pool of 5000 patients, a centre-stratified random sample of 550 patients was drawn, of which 394 were contacted. From those who were contacted, the first 192 persons who responded were enrolled, 189 completed all questionnaires and 185 came for an interview. Respondents and non-respondents were compared and no clinically or statistically significant differences were found between the two groups on socio-demographic characteristics.

### Measurement

#### Patient generated index

The PGI is an individualized measure of HRQL that was administered in three stages. In the first stage, patients were asked to identify up to five of the most important areas of their lives affected by MS. In the second stage, patients were asked to rate how badly affected they were in each of the selected areas on a scale of 0 to 10, where 0 was the worst they can imagine and 10 exactly as they would like to be. A sixth box was provided to rate all other health or non-health related areas. In the third stage, they were given twelve spending “points” or “tokens” to distribute among the areas identified. The tokens that they allocated to each area represented the relative importance of potential improvements in the chosen area. The more tokens a patient spent for an area, the more important that area was. The less tokens a patient spent, the less important that area was. The rating for each area was multiplied by the proportion of “points” for that area, which were then summed together to produce an index from 0 to 100 [49]. For ease of comparison with the utility measures, PGI scores in this study were presented on a scale from 0 to 1.

#### EQ-5D

The EQ-5D is a generic preference-based measure of HRQL that consists of two parts [50,51]. The first part includes 5 separate domains; mobility, capacity for self-care, conduct of usual activities, pain/discomfort and anxiety/depression. Each domain has 3 levels: no problems, some problems, extreme problems. The second part consists of a Visual Analogue Scale (EQVAS) to measure self-perceived health on a vertical scale from 0 to 100, where 0 is the worst imaginable health state, and 10 is the best imaginable health state. The EQ-5D defines 243 health states, and has a range from −0.6 to 1.0.

#### SF-6D

The SF-6D is a generic preference-based measure derived from the SF-36 Health Survey (or RAND-36) [23,39]. The SF-6D has 6 domains: physical functioning, role limitation, social functioning, pain, mental health and vitality. Each domain has between 4 and 6 levels. The index defines 18 000 health states, and has a range from 0.3 to 1.0.

### Procedure

**Figure 1:**
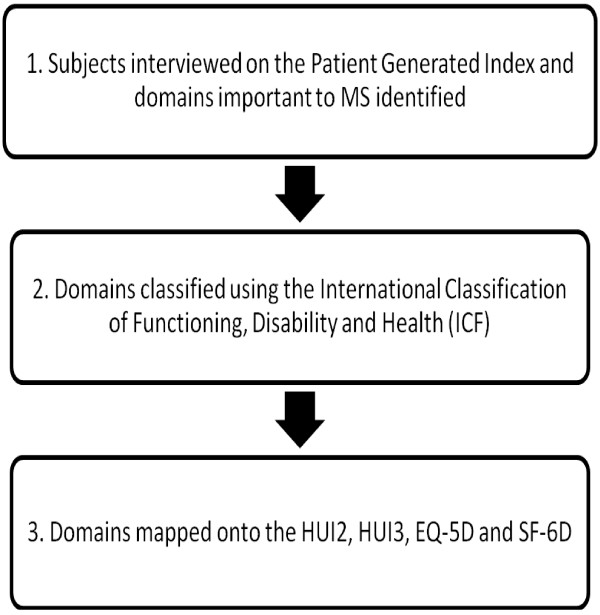
Flowchart of the study procedure.

Figure 1 presents a flowchart of the study procedure.

Subjects were first interviewed on an individualized measure of QOL, the PGI [49]. The domains identified with the PGI were then classified and grouped together using the World Health Organization’s International Classification of Functioning, Disability and Health (ICF) [52] independently by four raters. This methodology followed closely that conducted by Mayo et al [53], which evaluated the extent to which HRQL measures captured constructs beyond symptoms and function. The ICF provided a coding framework and standardized description of health related problems at the level of body structure/function (e.g. fatigue, cognition), activity (e.g. dressing, feeding, walking) and participation (e.g. school, work). These levels are also known as impairments, activity limitations and participation restrictions, respectively. Any discrepancies between raters were resolved by discussion.

Last, the domains were mapped onto the HUI2, HUI3, EQ-5D and SF-6D which had been previously mapped to the ICF [53]. The extent to which these utility measures captured domains important to patients with MS was qualitatively appraised.

### Data analysis

We had data on hand for the PGI, the EQ-5D and the SF-6D (derived from the RAND-36). As all three measures were administered on the same individual, generalized estimating equations (GEE) was used to adjust the variance for the clusters of outcome within persons. The advantage of using GEE, as opposed to the paired *t*-test, was that it allowed for simultaneous assessment and correlation among all 3 measures. The regression coefficients produced in the model were estimates of the difference between measures (with 95% CI) adjusted for the correlation among data points. An effect size (ES) was then calculated using the t-statistic, which was equal to the adjusted regression coefficient divided by its SE.

## Results

A total of 185 persons with MS were interviewed on the PGI. The sample was relatively young (mean age 43) and predominantly female. Both men and women had mild disability with a median Expanded Disability Status Scale (EDSS) score of 2. The average number of years since diagnosis was 6 years, and 59% of the sample was on Disease Modifying Therapies. Demographic and clinical characteristics are presented in Table 1.

**Table 1:** Demographic and clinical characteristics of sample (n = 185)

**Characteristics**	**Mean (SD) or N (%)**
Age (y)	42.8 (10.0)
Women/Men	137/48 (74/26)
Definite MS/CIS	170/15 (92/8)
Year since diagnosis	6.2 (3.6)
EDSS, median (IQR)	2.0 (1.0 - 3.5)
On DMT/Not on DMT/No information	110/19/56 (59/10/30)
Patient Generated Index*	0.50 (0.25)
EQ-5D**	0.69 (0.18)
SF-6D***	0.69 (0.13)

SD, standard deviation; N, number; CIS, Clinically Isolated Syndrome; EDSS, Expanded Disability Status Scale; IQR, Inter-quartile range; DMT, Disease Modifying Therapies.

*Transformed to a scale from 0 to 1, higher scores are better (1 = perfect QOL).

**Measured on a scale from −0.4 to 1, higher scores are better (1 = perfect health).

***Measured on a scale from 0.3 to 1, higher scores are better (1 = perfect health).

Table 2 presents the top 10 domains that patients identified to be the most affected by their MS. These areas were, work (62%), fatigue (48%), sports (39%), social life (28%), relationships (23%), walking/mobility (22%), cognition (21%), balance (14%), housework (12%) and mood (11%). The mean impact score for each domain (from 0 to 10) ranged from 3.9 to 5.0. In terms of the mean number of points spent for each domain, patients spent the most points (4.3) to improve their relationships, followed by fatigue (3.8) and then walking (mean 3.6).

**Table 2:** Top 10 domains identified by subjects using the Patient Generated Index

**Domain**	**Proportion of subjects reporting problem**	**Degree to which subjects are affected**	**Number of tokens spent**
	**N (%)**	Mean (SD)*	Mean (SD)**
School/Work	114 (62)	4.2 (3.4)	1.7 (2.0)
Fatigue	88 (48)	4.5 (2.2)	3.8 (2.7)
Sports	73 (39)	4.1 (2.6)	2.9 (2.4)
Social life	52 (28)	4.7 (2.4)	1.8 (2.6)
Relationships	43 (23)	4.8 (3.4)	4.3 (2.6)
Walking	41 (22)	3.9 (2.5)	3.6 (2.5)
Cognition	39 (21)	4.7 (2.1)	2.8 (2.2)
Balance	25 (14)	5.0 (2.3)	2.5 (3.3)
Housework	23 (12)	4.8 (2.1)	1.3 (1.0)
Mood	21 (11)	4.6 (2.4)	3.4 (2.6)

*Scored out of 10, higher is better (not affected).

**Scored out of 12, higher indicates that the domain was more important.

Table 3 presents the results for the mapping of the 10 domains identified by MS patients against the HUI2, HUI3, EQ-5D and the SF-6D. School/work was found in the EQ-5D and SF-6D but not in the HUI2 or HUI3. Fatigue was found in the SF-6D but not in the EQ-5D or the HUI measures. Sports which was the third most frequently reported domain, was only found in the SF-6D and HUI2. Social life was included in the EQ-5D and the SF-6D, but not in the HUI measures. Cognition was found in the HUI measures, but not in the EQ-5D or the SF-6D. Housework was included in the EQ-5D and the SF-6D, but not in the HUI2 or HUI3. Relationships and balance were not included in any of the utility measures. Mood was the only domain that was included in all of the measures.

**Table 3:** The domains identified by MS subjects compared with items in generic utility measures

**Measure**	**HUI2**	**HUI3**	**EQ-5D**	**SF-6D**
**Construct**	**Health status & HRQL **[35]**,**[36]	**Health status & HRQL **[36]**,**[37]	**HRQL **[38]	**Health status **[39]
**MS Domains**				
School/Work	N	N	Y	Y
Fatigue	N	N	N	Y
Sports	Y	N	N	Y
Social life	N	N	N	Y
Relationships	N	N	N	N
Cognition	Y	Y	N	N
Walking	Y	Y	Y	N
Housework	N	N	Y	Y
Balance	N	N	N	N
Mood*	Y	Y	Y	Y
Total Yes (out of 10)	4	3	4	6
**Not MS Domains**				
Pain	Y	Y	Y	Y
Self-care	Y	N	Y	Y
Vision	Y	Y	N	N
Hearing	Y	Y	N	N
Manual dexterity	N	Y	N	N
Speech	Y	Y	N	N
Fertility	Y	N	N	N

MS Domains ordered from the largest to the smallest proportion of people with MS who identified that domain.

Y, Yes; N, No; HUI2, Health Utilities Index Mark 2; HUI3, Health Utilities Index Mark 3; SF-6D, EQ-5D, EuroQol-5D; Short-Form 6D.

*In the HUI3 this was happiness.

The SF-6D included the most number of domains (6 domains) important to people with MS, followed by the EQ-5D (4 domains) and the HUI2 (4 domains), and then the HUI3 (3 domains).

The generic utility measures included domains that were not identified to be important by the sample, such as pain, self-care, vision, hearing, manual dexterity, speech and fertility.

**Figure 2:**
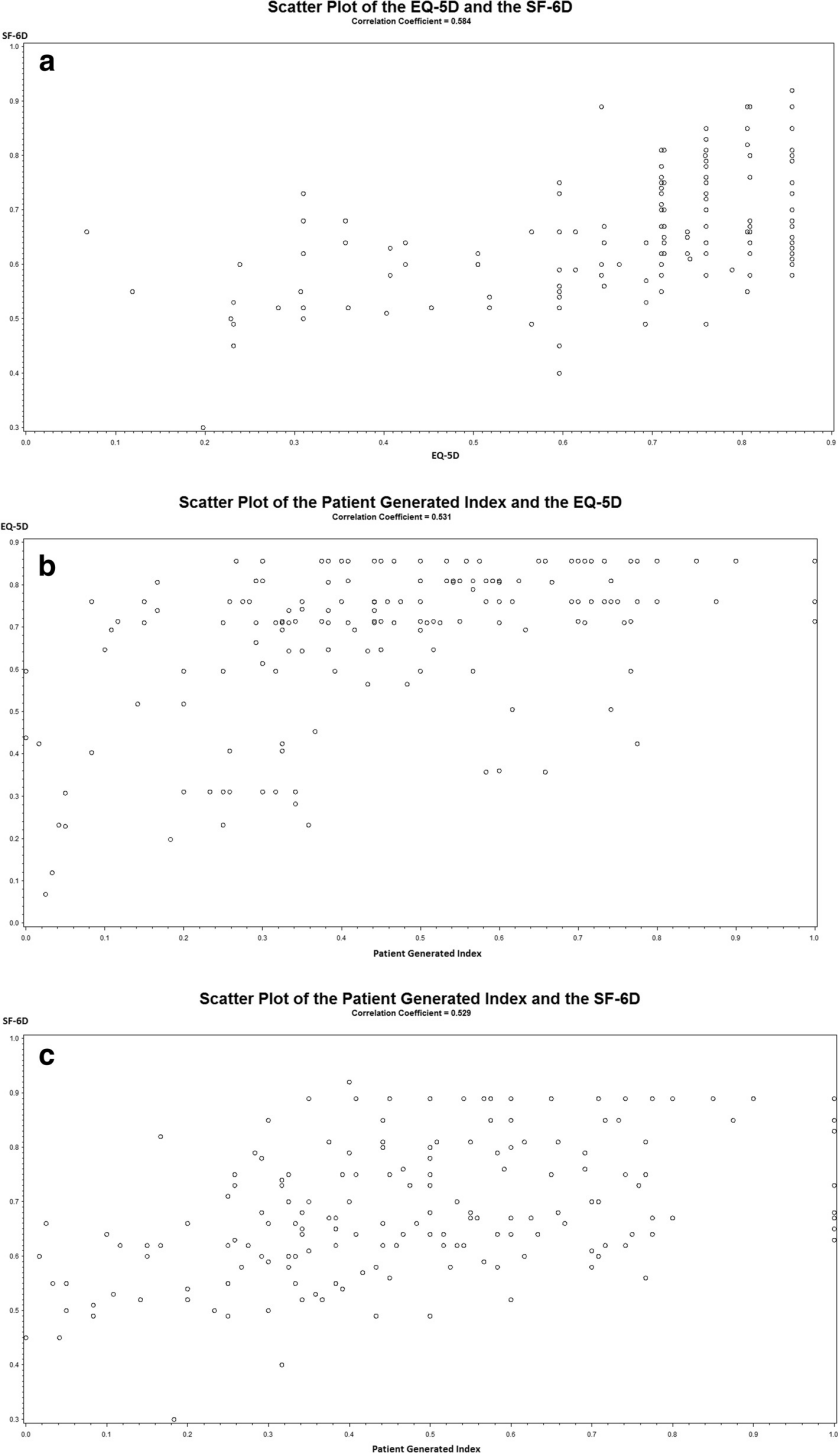
**Relationship between the EQ-5D, the SF-6D and the Patient Generated Index. a**: Scatter plot of the relationship between the EQ-5D and the SF-6D. **b**: Scatter plot of the relationship between the Patient Generated Index and the EQ-5D. **c**: Scatter plot of the relationship between the Patient Generated Index and the SF-6D.

The correlation between the SF-6D and the EQ-5D was 0.58. As demonstrated in Figure 2a, although the relationship between the measures was somewhat linear, discrepancies in scores between the two measures was evident. At the upper end of the scales, a number of individuals who had utility scores of 0.85 on the EQ-5D had scores as low as 0.6 on the SF-6D. A clinically meaningful difference on utility measures is 0.03, indicating that the difference in scores between the two utility measures was important. Discrepancies were also observed at the lower end of the scale, where an individual with a score of 0.12 on the EQ-5D had a score of 0.55 on the SF-6D.

The correlation between the PGI and the EQ-5D was 0.53. As presented in Figure 2b there were important discrepancies in scores between the two measures. Several individuals with very low scores on the PGI (as low as 0.1) had very high scores on the EQ-5D (as high as 0.8). For many individuals, there was also a mismatch between scores obtained using the PGI and those obtained with the EQ-5D (i.e. individuals with scores as low as 0.1 on the PGI had scores of 0.8 on the EQ-5D). Pearson’s correlation between the PGI and the SF-6D was 0.53. Similar to what was observed for the EQ-5D; there were discrepancies in scores between the 2 measures, particularly towards the lower end of the scales (Figure 2c).

**Figure 3:**
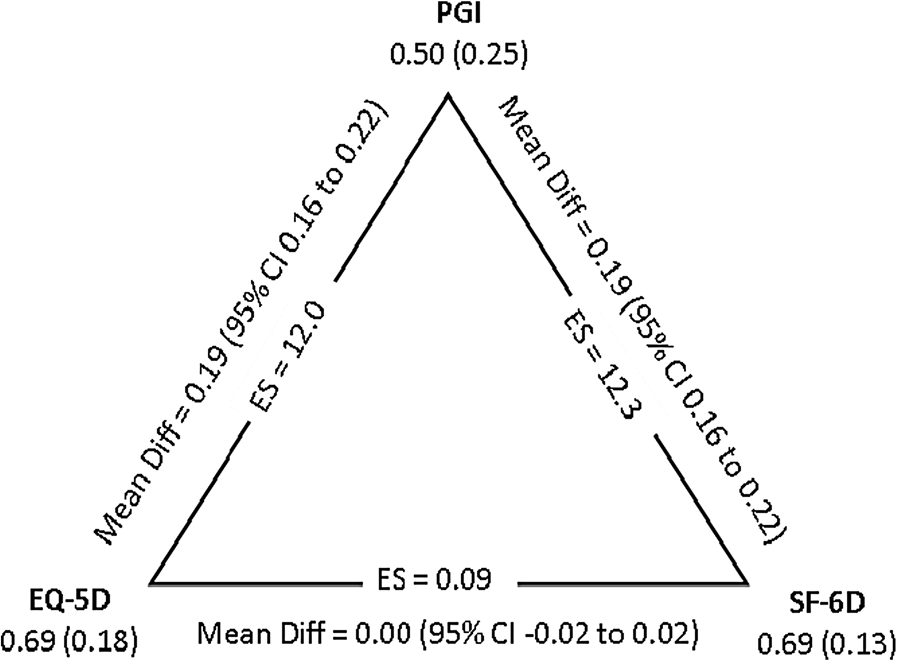
Mean and standard deviation values for the PGI, EQ-5D and SF-6D, with differences and 95% CI calculated using generalized estimating equations.

The impact of a mismatch between domains provided in the generic utility measures and those that are important to people with MS is illustrated by the total scores of the measures. As seen in Figure 3, the mean and standard deviation (SD) for the PGI, EQ-5D and the SF-6D were 0.50 (SD 0.25), 0.69 (SD 0.18) and 0.69 (SD 0.13), respectively. The magnitude of difference between the PGI and the 2 utility measures was 0.19 (95% CI 0.16 to 0.22) with ES equal to 12.

**Figure 4:**
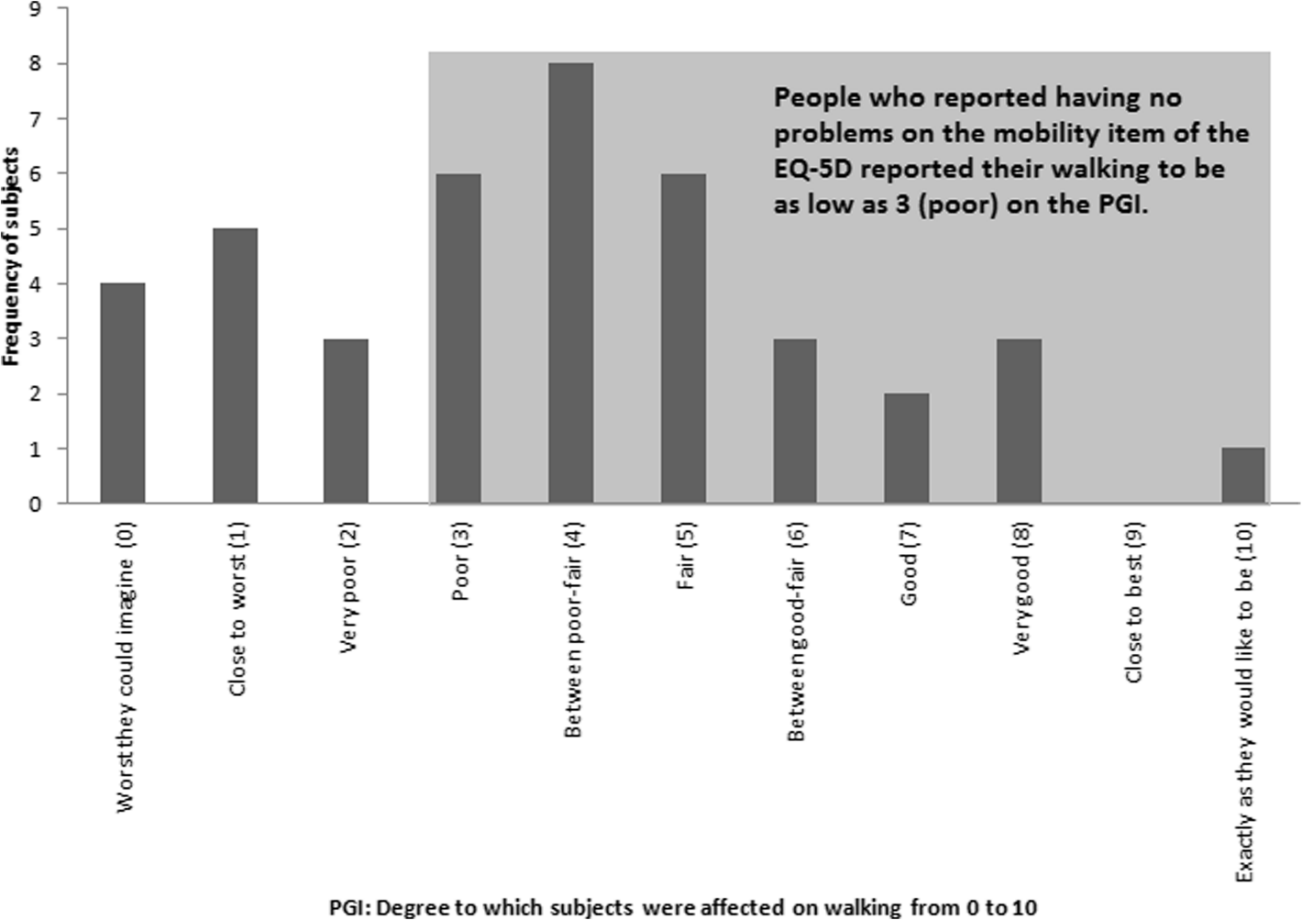
Frequency and distribution of PGI scores on the degree to which walking was affected from 0 (worst they can imagine) to 10 (exactly as they would like to be).

This mismatch was also present at the item level. A total of 41 subjects (22% of the sample) reported walking to be an important aspect of their QOL. The distribution of scores on the degree to which walking was affected for these subjects is presented in Figure 4. The impact was measured on a scale from 0 to 10 on the PGI, where 0 was the worst they could imagine and 10 was exactly as they would like to be. These scores were compared with the responses on the EQ-5D mobility item. 12 subjects out of 41 reported having no problems with walking on the EQ-5D. These people were expected to have a score of 10 on the PGI. Only 1 person reported a score of 10 on the PGI. All other subjects reported scores lower than this, scores as low as 3 (poor).

## Discussion

In this study, subjects with MS were interviewed on an individualized measure to evaluate the impact of the disease on their QOL. The results of the interview generated a list of domains that were most important to the QOL of persons with MS. The domains identified were work, fatigue, sports, social life, relationships, walking, cognition, balance, housework and mood. These were then mapped onto generic utility measures to estimate the extent to which they captured domains that were important to persons with MS.

There was no one generic utility measure that captured all of the domains important to persons with MS. For example, fatigue, which affects 75 to 90% of patients with MS [54-57] was not included in the EQ-5D or the HUI measures. Walking, another commonly reported symptom was not found in the SF-6D. Cognition was not found in the EQ-5D or the SF-6D. Work, sports, and social life were not found in the HUI2 or HUI3. This was not surprising as the HUI measures were developed with the intention of evaluating ‘within-the-skin’ experiences that excluded social interaction [58-60]. Balance and relationships were not included in any of the utility measures.

The generic utility measures were clearly missing domains that were important to people with MS. Out of the 10 domains that persons with MS identified as being central to their QOL, only 3 of them were included in the HUI2, 4 were included in the HUI3, 4 were included in the EQ-5D and 6 were included in the SF-6D. Furthermore, the generic utility measures included several domains that were not important to persons which were sampled in the study, such as pain, self-care, hearing and manual dexterity.

To tackle the issue of lack of content validity, one emerging area of interest in the literature is the development of disease specific “bolt-ons” or dimension extensions to generic utility measures [51]. Another emerging area of interest is the development of disease-specific utility measures, which have been developed for stroke [61], pulmonary hypertension [62], asthma [63], rhinitis [64], urinary incontinence [65] and erectile dysfunction [66]. Recently, Versteegh et al. [67] derived a MS specific utility measure from the Multiple Sclerosis Impact Scale-29 (MSIS-29) using Rasch analysis. The authors selected 8 out of 29 items from the original questionnaire. Some important dimensions such as social life, work and mood were included while others such as walking, sports and physical fatigue were omitted.

There are several potential benefits to using disease specific utility measures in clinical and cost-effectiveness research. First, disease specific utility measures are designed to include domains that are specific to a disease, and therefore, are likely to be more sensitive to smaller change over time than generic measures. Second, not only do these measures provide descriptive information on the various dimensions of health, but also provide a value for each one, thus allowing trade-offs to be made between the domains. Disease-specific utility measures serve the potential to overcome one of the challenges associated with disease specific health profiles - that domains cannot be combined into a single index, which makes it difficult to conclude whether an intervention was effective or not. For example, if a treatment has a positive effect on physical health but a negative one on mental health, unless we know the relative importance attached to each domain, it is impossible to determine whether the intervention resulted in a net improvement or decline in QOL/HRQL. Furthermore, disease-specific utility measures can be used to calculate QALYs and make decisions on the cost-effectiveness of different treatments in MS.

A clinician reported outcome (ClinRO) is an assessment of the status of a patient’s health condition that is made by an observer with professional training (i.e. clinician) [69]. ClinRO are commonly used for endpoints that cannot be directly measured by the patient (e.g. EDSS to quantify level of disability in MS). An observer-reported outcome (ObsRO) is an assessment that is made by an observer without professional training (i.e. non-clinician observer such as a teacher or caregiver) [69]. This type of evaluation is typically used when the patient is unable to self-report. A patient reported outcome (PRO) is any report of the status of a patient’s health condition that comes directly from the patient, without interpretation of the patient’s response by a clinician or other observer (e.g. symptoms, QOL, HRQL) [68,69]. PROs play a complementary role in outcome assessment by providing evidence on the benefit or harm of a treatment from the patient’s perspective. Utility measures are one type of PRO. In outcome assessment, utility measures not only provide information on the benefits and harms of a treatment, but are also useful for economic applications by producing QALYS. This information can provide policy and decision makers with a means of evaluating the costs and cost-effectiveness of different treatment options for a health condition.

The first step in evaluating the validity of scores produced by a PRO is an assessment of content validity, before any other forms of validity (i.e. construct validity) are undertaken. Content validity of a PRO can be judged only by the individuals or populations being assessed (i.e. the patients themselves). The global aim of this study was to address this very question of whether generic utility measures captured domains that were important or relevant to people with MS. The results of this study suggest that many important domains in MS are not captured by generic utility measures, therefore questioning the content validity of such measures in MS. This in turn, adds doubt to the interpretability or meaningfulness of scores produced by these measures for this population.

It is important to target measures to people to ensure that the impact of a disease and its treatment are adequately and reliably captured in a clinical trial [70,71]. If a PRO includes domains that are not impacted upon by the disease or its treatment, it will not be able to capture clinically meaningful change. By targeting to the disease, measures are more likely to be sensitive to small but important clinical changes. Furthermore, the ability of PROs to detect small changes is important in determining the statistical power or the necessary sample size required for a clinical trial [72].

The results of our study revealed that the commonly used 4 generic utility measures (HUI2, HUI3, EQ-5D and SF-6D) do not capture the majority of domains important to MS. Among these generic measures, the SF-6D captured the most number of domains (6 domains) that were important to MS. Our findings suggest that the SF-6D, compared to the other generic utility measures, may be the most appropriate one to use in MS. The PGI index can be used to evaluate the clinical effectiveness of different interventions in MS. However, because the PGI was not developed using multi-attribute utility theory (hence is not a utility measure); it cannot be used for cost-utility analysis.

Ideas for future directions that build directly from this work are the use of MS specific “bolt-on” items or dimensions to generic utility measures [73]. This study has identified potential items important to MS, such as fatigue that can be used as add-ons to existing generic utility measures. Other areas of potential research that can build directly from this work are the development of an MS specific utility measure that will only include dimensions pertinent to the disease.

A particular feature of this study is that we purposely sampled people with MS diagnosed in the era of Magnetic Resonance Imaging (MRI) technology and availability of disease modifying drugs [48]. As these are the people who are faced with treatment decisions, a method of valuing changes on the most important domains of QOL affected by MS would be the most relevant for this population.

## Conclusions

Generic utility measures are designed to include a common set of dimensions that most people will value highly, therefore underrepresenting those dimensions that may be specific to a particular disease. Although the generic utility measures included certain items that were important to people with MS, there were several that were missing. An important consequence of this mismatch was that values of QOL derived from the PGI were importantly and significantly lower than those estimated using any of the generic utility measures. This could have a substantial impact for evaluating the effect of interventions in people with MS. The overestimation in scores obtained with utility measures may not have an impact at the start of a clinical trial, but they will have an impact at follow-up. If scores are high at baseline, there will likely be no room for improvement on the scale, resulting in the false conclusion that the treatment group did not change post-treatment. When in reality, the treatment may have had a positive effect but the measure being administered was not able to detect this. Then the difference between the treatment and control group (assuming the control also does not change), would be zero. In addition, an intervention that is in fact beneficial to fatigue, for example, would also risk not to show change on a generic measure because this item was not included. When choosing the right outcome measure for an intervention, it is essential to choose one with items that can or should be affected by the intervention. Given that the MS specific items do impact on QOL, not including these items would result in a false estimate of QALYs and bias the evaluation of the cost-effectiveness of interventions in MS.

## Abbreviations

MS: Multiple Sclerosis; HUI2: Health Utilities Index Mark 2; HUI3: Health Utilities Index Mark 3; EQ-5D: EuroQol-5D; SF-6D: Short-Form-6D; CADTH: Canadian Agency for Drugs and Technologies in Health; QOL: Quality of life; HRQL: Health-related quality of life; QALY: Quality-Adjusted Life Year; MRI: Magnetic resonance imaging; PGI: Patient Generated Index; ICF: International Classification of Functioning, Disability and Health; ES: Effect size; EDSS: Expanded Disability Status Scale.

## Competing interests

The authors declare that they have no competing interests.

## Authors’ contribution

NM was the principal investigator of the study and AK collected the data. Both authors contributed to writing the article, data analysis and interpretation. Both authors read and approved the final manuscript.

## Authors’ information

AK is a physiotherapist and a PhD Candidate in Rehabilitation Sciences at the School of Physical and Occupational Therapy, McGill University. NM is a James McGill Professor in the Department of Medicine and the School of Physical and Occupational Therapy, McGill University.

## References

[B1] Noseworthy JH Lucchinetti C Rodriguez M Weinshenker BG Multiple sclerosis N Engl J Med 2000 343 938 952 10.1056/NEJM200009283431307 11006371

[B2] Burks J Johnson K: Multiple S Diagnosis, Medical Management, and Rehabilitation 2000 New York: Demos Medical

[B3] Miller DM Moore SM Fox RJ Atreja A Fu AZ Lee JC Web-based self-management for patients with multiple sclerosis: a practical, randomized trial Telemed J E-Health 2011 17 5 13 10.1089/tmj.2010.0133 21214498 PMC3064874

[B4] Barlow J Turner A Edwards R Gilchrist M A randomised controlled trial of lay-led self-management for people with multiple sclerosis Patient Educ Couns 2009 77 81 89 10.1016/j.pec.2009.02.009 19321290

[B5] Bombardier CH Cunniffe M Wadhwani R Gibbons LE Blake KD Kraft GH The Efficacy of Telephone Counseling for Health Promotion in People With Multiple Sclerosis: A Randomized Controlled Trial Arch Phys Med Rehabil 2008 89 10 1849 1856 10.1016/j.apmr.2008.03.021 18929012

[B6] McAuley E Motl RW Morris KS Hu L Doerksen SE Elavsky S Enhancing physical activity adherence and well-being in multiple sclerosis: a randomised controlled trial Mult Scler 2007 13 652 659 10.1177/1352458506072188 17548446

[B7] Grossman P Kappos L Gensicke H D'Souza M Mohr DC Penner IK MS quality of life, depression, and fatigue improve after mindfulness training: a randomized trial Neurology 2010 75 1141 1149 10.1212/WNL.0b013e3181f4d80d 20876468 PMC3463050

[B8] Forman AC Lincoln NB Evaluation of an adjustment group for people with multiple sclerosis: a pilot randomized controlled trial Clin Rehabil 2010 24 211 221 10.1177/0269215509343492 20026575

[B9] Cosio D Jin L Siddique J Mohr DC The effect of telephone-administered cognitive-behavioral therapy on quality of life among patients with multiple sclerosis Ann Behav Med 2011 41 227 234 10.1007/s12160-010-9236-y 21069585 PMC3108488

[B10] Kavia RB De RD Constantinescu CS Stott CG Fowler CJ Randomized controlled trial of Sativex to treat detrusor overactivity in multiple sclerosis Mult Scler 2010 16 1349 1359 10.1177/1352458510378020 20829244

[B11] Moller F Poettgen J Broemel F Neuhaus A Daumer M Heesen C HAGIL (Hamburg Vigil Study): A randomized placebo-controlled double-blind study with modafinil for treatment of fatigue in patients with multiple sclerosis Mult Scler 2011 17 8 1002 1009 10.1177/1352458511402410 21561959

[B12] Freeman JA Thompson AJ Fitzpatrick R Hutchinson M Miltenburger C Beckmann K Interferon-beta1b in the treatment of secondary progressive MS: impact on quality of life Neurology 2001 57 1870 1875 10.1212/WNL.57.10.1870 11723278

[B13] Rudick RA Miller D Hass S Hutchinson M Calabresi PA Confavreux C Health-related quality of life in multiple sclerosis: effects of natalizumab Ann Neurol 2007 62 335 346 10.1002/ana.21163 17696126

[B14] Fox RJ Miller DH Phillips JT Hutchinson M Havrdova E Kita M Placebo-controlled phase 3 study of oral BG-12 or glatiramer in multiple sclerosis N Engl J Med 2012 367 1087 1097 10.1056/NEJMoa1206328 22992072

[B15] Zamboni P Menegatti E Galeotti R Malagoni AM Tacconi G Dall'Ara S The value of cerebral Doppler venous haemodynamics in the assessment of multiple sclerosis J Neurol Sci 2009 282 21 27 10.1016/j.jns.2008.11.027 19144359

[B16] Al-Omari MH Rousan LA Internal jugular vein morphology and hemodynamics in patients with multiple sclerosis Int Angiol 2010 29 115 120 20351667

[B17] Baracchini C Perini P Calabrese M Causin F Rinaldi F Gallo P No evidence of chronic cerebrospinal venous insufficiency at multiple sclerosis onset Ann Neurol 2011 69 90 99 10.1002/ana.22228 21280079

[B18] Centonze D Floris R Stefanini M Rossi S Fabiano S Castelli M Proposed chronic cerebrospinal venous insufficiency criteria do not predict multiple sclerosis risk or severity Ann Neurol 2011 70 51 58 21786298 10.1002/ana.22436

[B19] Zivadinov R Marr K Cutter G Ramanathan M Benedict RH Kennedy C Prevalence, sensitivity, and specificity of chronic cerebrospinal venous insufficiency in MS Neurology 2011 77 138 144 10.1212/WNL.0b013e318212a901 21490322

[B20] The World Health Organization Quality of Life assessment (WHOQOL): position paper from the World Health Organization Soc Sci Med 1995 41 1403 1409 8560308 10.1016/0277-9536(95)00112-k

[B21] Ware JE Jr Standards for validating health measures: definition and content J Chronic Dis 1987 40 473 480 10.1016/0021-9681(87)90003-8 3298292

[B22] Breslow L A quantitative approach to the World Health Organization definition of health: physical, mental and social well-being Int J Epidemiol 1972 1 347 355 10.1093/ije/1.4.347 4669083

[B23] Brazier J Roberts J Deverill M The estimation of a preference-based measure of health from the SF-36 J Health Econ 2002 21 271 292 10.1016/S0167-6296(01)00130-8 11939242

[B24] World Health Organization Glossary of Terms Used in the “Health For All” Series 1984 Ref Type: Report23619467

[B25] Kind P Fayers P, Hays D Values and valuation in the measurement of HRQoL Assessing quality of life in clinical trials 2005 2 New York: Oxford University Press Inc 391 404

[B26] Guyatt GH Feeny DH Patrick DL Measuring health-related quality of life Ann Intern Med 1993 118 622 629 10.7326/0003-4819-118-8-199304150-00009 8452328

[B27] Feeny DH Torrance GW Incorporating utility-based quality-of-life assessment measures in clinical trials. Two examples Med Care 1989 27 S190 S204 10.1097/00005650-198903001-00016 2522159

[B28] Torrance GW Utility approach to measuring health-related quality of life J Chronic Dis 1987 40 593 603 10.1016/0021-9681(87)90019-1 3298297

[B29] Torrance GW Measurement of health state utilities for economic appraisal J Health Econ 1986 5 1 30 10.1016/0167-6296(86)90020-2 10311607

[B30] Feeny D Fayers P, Hays D Preference-based measures: utility and quality-adjusted life years Assessing quality of life in clinical trials 2005 2 New York: Oxford University Press Inc 405 429

[B31] Brazier J Ratcliffe J Salomon JA Tsuchiya A Measuring and valuing health benefits for economic evaluation 2007 New York: Oxford University Press Inc.

[B32] Kind P Lafata JE Matuszewski K Raisch D The use of QALYs in clinical and patient decision-making: issues and prospects Value Health 2009 12 Suppl 1 S27 S30 19250128 10.1111/j.1524-4733.2009.00519.x

[B33] Hawthorne G Richardson J Measuring the value of program outcomes: a review of multiattribute utility measures Expert Rev Pharmacoecon Outcomes Res 2001 1 215 228 10.1586/14737167.1.2.215 19807409

[B34] Flanagan JC Measurement of quality of life: current state of the art Arch Phys Med Rehabil 1982 63 56 59 6460487

[B35] Torrance GW Feeny DH Furlong WJ Barr RD Zhang Y Wang Q Multiattribute utility function for a comprehensive health status classification system. Health Utilities Index Mark 2 Med Care 1996 34 702 722 10.1097/00005650-199607000-00004 8676608

[B36] Horsman J Furlong W Feeny D Torrance G The Health Utilities Index (HUI): concepts, measurement properties and applications Health Qual Life Outcomes 2003 1 54 10.1186/1477-7525-1-54 14613568 PMC293474

[B37] Feeny D Furlong W Torrance GW Goldsmith CH Zhu Z DePauw S Multiattribute and single-attribute utility functions for the health utilities index mark 3 system Med Care 2002 40 113 128 10.1097/00005650-200202000-00006 11802084

[B38] Gudex C Kind P, Brooks R, Rabin R The descriptive system of the EuroQOL instrument EQ-5D concepts and methods: a developmental history 2005 Dordrecht: Springer 19 33

[B39] Brazier J Usherwood T Harper R Thomas K Deriving a preference-based single index from the UK SF-36 Health Survey J Clin Epidemiol 1998 51 1115 1128 10.1016/S0895-4356(98)00103-6 9817129

[B40] Ahmed S Mayo NE Wood-Dauphinee S Hanley JA Cohen SR Using the Patient Generated Index to evaluate response shift post-stroke Qual Life Res 2005 14 2247 2257 10.1007/s11136-005-8118-4 16328904

[B41] Goldstein RS Gort EH Stubbing D Avendano MA Guyatt GH Randomised controlled trial of respiratory rehabilitation Lancet 1994 344 1394 1397 10.1016/S0140-6736(94)90568-1 7968075

[B42] Lacasse Y Wong E Guatt GH Joyce CR, O'Boyle CA, McGee H Individualising questionnaires Individual quality of life: Approaches to conceptualization and assessment 1999 Amsterdam: Harwood Academic Publishers 87 103

[B43] Simpson K Killian K McCartney N Stubbing DG Jones NL Randomised controlled trial of weightlifting exercise in patients with chronic airflow limitation Thorax 1992 47 70 75 10.1136/thx.47.2.70 1549826 PMC463573

[B44] Wijkstra PJ Van AR Kraan J Otten V Postma DS Koeter GH Quality of life in patients with chronic obstructive pulmonary disease improves after rehabilitation at home Eur Respir J 1994 7 269 273 10.1183/09031936.94.07020269 8162979

[B45] Kettis-Lindblad A Ring L Widmark E Bendtsen P Glimelius B Patients’ and doctors’ views of using the schedule for individual quality of life in clinical practice J Support Oncol 2007 5 281 287 17624053

[B46] Detmar SB Muller MJ Schornagel JH Wever LD Aaronson NK Health-related quality-of-life assessments and patient-physician communication: a randomized controlled trial JAMA 2002 288 3027 3034 10.1001/jama.288.23.3027 12479768

[B47] Patel KK Veenstra DL Patrick DL A review of selected patient-generated outcome measures and their application in clinical trials Value Health 2003 6 595 603 10.1046/j.1524-4733.2003.65236.x 14627066

[B48] Mayo N Setting the agenda for multiple sclerosis rehabilitation research Mult Scler 2008 14 1154 1156 10.1177/1352458508096567 18952830

[B49] Ruta DA Garratt AM Leng M Russell IT MacDonald LM A new approach to the measurement of quality of life. The Patient-Generated Index Med Care 1994 32 1109 1126 10.1097/00005650-199411000-00004 7967852

[B50] Dolan P Modeling valuations for EuroQol health states Med Care 1997 35 1095 1108 10.1097/00005650-199711000-00002 9366889

[B51] Shaw JW Johnson JA Coons SJ US valuation of the EQ-5D health states: development and testing of the D1 valuation model Med Care 2005 43 203 220 10.1097/00005650-200503000-00003 15725977

[B52] International Classification of Functioning Disability and Health (ICF) 2001 Geneva: World Health Organization

[B53] Mayo NE Moriello C Asano M van der Spuy S Finch L The extent to which common health-related quality of life indices capture constructs beyond symptoms and function Qual Life Res 2011 20 621 627 10.1007/s11136-010-9801-7 21108008

[B54] Krupp LB Pollina DA Mechanisms and management of fatigue in progressive neurological disorders Curr Opin Neurol 1996 9 456 460 10.1097/00019052-199612000-00011 9007405

[B55] Fisk JD Pontefract A Ritvo PG Archibald CJ Murray TJ The impact of fatigue on patients with multiple sclerosis Can J Neurol Sci 1994 21 9 14 8180914

[B56] Freal JE Kraft GH Coryell JK Symptomatic fatigue in multiple sclerosis Arch Phys Med Rehabil 1984 65 135 138 6703889

[B57] Murray TJ Amantadine therapy for fatigue in multiple sclerosis Can J Neurol Sci 1985 12 251 254 3902184 10.1017/s0317167100047107

[B58] Feeny D Furlong W Barr RD Torrance GW Rosenbaum P Weitzman S A comprehensive multiattribute system for classifying the health status of survivors of childhood cancer J Clin Oncol 1992 10 923 928 1316952 10.1200/JCO.1992.10.6.923

[B59] Feeny D Furlong W Boyle M Torrance GW Multi-attribute health status classification systems. Health Utilities Index Pharmacoeconomics 1995 7 490 502 10.2165/00019053-199507060-00004 10155335

[B60] Feeny D Torrance GW Furlong W Spilker B Health utilities index Quality of Life and Pharmaeconomics in Clinicals Trials 1996 2 Philadelphia: Lippincott-Raven Publishers 239 252

[B61] Poissant L Mayo NE Wood-Dauphinee S Clarke AE The development and preliminary validation of a Preference-Based Stroke Index (PBSI) Health Qual Life Outcomes 2003 1 43 10.1186/1477-7525-1-43 14561225 PMC222917

[B62] McKenna SP Ratcliffe J Meads DM Brazier JE Development and validation of a preference based measure derived from the Cambridge Pulmonary Hypertension Outcome Review (CAMPHOR) for use in cost utility analyses Health Qual Life Outcomes 2008 6 65 10.1186/1477-7525-6-65 18718016 PMC2546377

[B63] Revicki DA Leidy NK Brennan-Diemer F Sorensen S Togias A Integrating patient preferences into health outcomes assessment: the multiattribute Asthma Symptom Utility Index Chest 1998 114 998 1007 10.1378/chest.114.4.998 9792568

[B64] Revicki DA Leidy NK Brennan-Diemer F Thompson C Togias A Development and preliminary validation of the multiattribute Rhinitis Symptom Utility Index Qual Life Res 1998 7 693 702 10097618 10.1023/a:1008860113818

[B65] Brazier J Czoski-Murray C Roberts J Brown M Symonds T Kelleher C Estimation of a preference-based index from a condition-specific measure: the King's Health Questionnaire Med Decis Making 2008 28 113 126 17641139 10.1177/0272989X07301820

[B66] Torrance GW Keresteci MA Casey RW Rosner AJ Ryan N Breton MC Development and initial validation of a new preference-based disease-specific health-related quality of life instrument for erectile function Qual Life Res 2004 13 349 359 15085907 10.1023/B:QURE.0000018482.71580.f2

[B67] Versteegh MM Leunis A Uyl-de Groot CA Stolk EA Condition-specific preference-based measures: benefit or burden? Value Health 2012 15 504 513 10.1016/j.jval.2011.12.003 22583461

[B68] Food US Drug Administration: Guidance for industry: Patient-reported outcome measures. Use in medical product development to support labeling claims Fed Regist 2009 74 65132 65133

[B69] Velentgas P Dreyer NA Wu AW Velentgas P, Dreyer NA, Nourjah P Outcome definition and measurement Developing a Protocol for Observational Comparative Effectiveness Research: A User's Guide. AHRQ Publication No. 12(13)-EHC099 2013 Rockville, MD: Agency for Healthcare Research and Quality 71 92 23469377

[B70] Hays RD Fayers P, Hays D Generic versus disease-targeted instruments Assessing quality of life in clinical trials 2005 2 New York: Oxford University Press Inc 3 8

[B71] Guyatt GH Bombardier C Tugwell PX Measuring disease-specific quality of life in clinical trials CMAJ 1986 134 889 895 3955482 PMC1490966

[B72] Mayo NE Bailar JC, Hoaglin DC Randomized trials and other parallel comparisons of treatments Medical Uses of Statistics 2009 3 Hoboken: John Wiley & Sons, Inc 51 89

[B73] Lin FJ Longworth L Pickard AS Evaluation of content on EQ-5D as compared to disease-specific utility measures Qual Life Res Epub ahead of print 10.1007/s11136-012-0207-6 22729670

